# Multiple pulmonary cavernous haemangiomas with concurrent primary pulmonary adenocarcinoma: a case report and literature review

**DOI:** 10.3389/fonc.2024.1353592

**Published:** 2024-01-29

**Authors:** Xiao Wang, Tao Ren, Hui You, Wenya Han, Jialong Guo, Meifang Wang

**Affiliations:** ^1^ Department of Pulmonary and Critical Care Medicine, Taihe Hospital, Hubei University of Medicine, Shiyan, Hubei, China; ^2^ Department of Thoracic Surgery, Taihe Hospital, Hubei University of Medicine, Shiyan, Hubei, China

**Keywords:** pulmonary cavernous haemangioma, multiple nodules, adenocarcinoma, video-assisted thoracic surgery, PET-CT

## Abstract

**Background:**

Cavernous haemangiomas (CHs) commonly occurred in the skin, subcutaneous tissue, muscles, and liver. Pulmonary cavernous haemangiomas (PCHs) are quite rare and usually present with nonspecific clinical symptoms. When lung cancer patients are complicated with pulmonary cavernous haemangiomas, radiologically, these haemangioma lesions can be easily misinterpreted as intrapulmonary metastases, potentially resulting in misdiagnosis, or missed diagnosis.

**Case presentation:**

The present study reported the case of a 53−year−old female patient with pulmonary adenocarcinoma coexisting with multiple PCHs. ^18^F−FDG-Positron emission tomography-computed tomography (PET-CT) showed an elevated glucose metabolism in the apicoposterior segment of the left upper lobe; however, the other nodules were not hypermetabolic. Percutaneous lung biopsy was performed on the nodule in the apicoposterior segment of the left upper lobe, which were diagnosed as primary adenocarcinoma. Some nodules in the upper left lobe underwent wedge resection by video-assisted thoracic surgery (VATS) and intraoperative frozen section identified as PCHs. Finally, the patient underwent lobectomy of the left upper lobe via VATS, cancerous nodule in the apicoposterior segment of the left upper lobe underwent genetic molecular testing of PCR−Sanger sequencing, suggested *EGFR* mutation, and patient received treatment with Osimertinib. During the 4−months follow−up, contrast−enhanced CT showed no recurrence of either disease. PCHs are rare benign tumours of the lung, which can lead to misdiagnosis due to their non-specific clinical symptoms and radiological features, especially when they coexist with lung cancer. PCHs is easily misunderstood as metastatic lung cancer, in this case, PET-CT can assist in differentiating benign from malignant. For the cases of early lung cancer complicated with PCHs, lung cancer can be surgically resected, and whether PCHs should be removed or not should be determined according to the size and distribution of the lesions.

## Introduction

PCHs are rare benign tumour of the lungs, originating from congenital pulmonary vascular malformations that give rise to direct arteriovenous communications, establishing a pulmonary circulatory shunt. Cavernous haemangiomas are predominantly localised in the skin, subcutaneous tissue, muscles, and liver. Their primary occurrence in the lungs is exceedingly uncommon (1). PCHs do not present with specific clinical symptoms and are easily misdiagnosed as other ailments, such as lung cancer, tuberculosis, or pulmonary cysts, during routine X-ray and CT examinations (2). When lung cancer is concomitant with PCHs, haemangioma lesions are prone to misidentification as metastatic foci of lung cancer, resulting in delayed treatment. This article offers a retrospective analysis of clinical data from a patient diagnosed with a rare case of bilateral diffuse pulmonary cavernous haemangioma concurrent with pulmonary adenocarcinoma, which were finally confirmed by surgical pathology in our hospital. We reviewed the clinical features, pathological histological characteristics, diagnosis, and treatment in conjunction with relevant literature for a better understanding of PCHs.

## Case report

**Figure 1:**
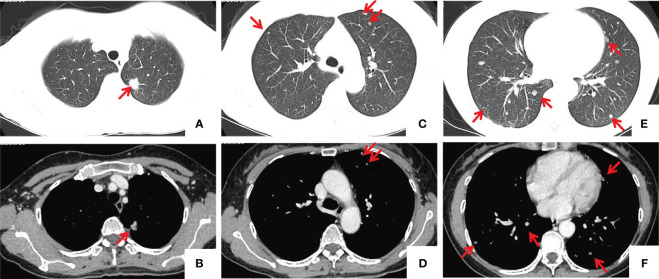
Chest CT images. **(A, B)** A nodule located in the apicoposterior segment of the left upper lobe, approximately 1.3x1.4 cm in size, exhibits spiculation and lobulation, presenting moderate heterogeneous enhancement and causing adjacent pleural traction. **(C–F)** Multiple nodules of varying sizes are present in the upper and lower lobes of both lungs (indicated by red arrows), with the larger ones measuring approximately 1.0 cm in diameter, showing mild enhancement. the mediastinal lymph nodes were normal.

A 53-year-old female patient was admitted to Taihe Hospital (Shiyan, China) due to a nodule in the upper lobe of the left lung during a routine physical examination. The patient had no respiratory symptoms and denied any other discomfort. The patient had no history of underlying diseases, including tuberculosis, cancer, hepatitis, diabetes, or connective tissue disease. On admission, physical examination revealed normal bilateral respiratory movement and tactile fremitus, and the patient had clear percussion notes and breath sounds. No dry or moist rales or pleural friction rubs were audible, the vital signs *etc.* were normal, there were no skin lesions, lymphadenopathy or splenomegaly. Contrast-enhanced computed tomography (CT) of Chest showed a 1.3x1.2-cm nodule in the apicoposterior segment of the left upper lobe (**Figures 1A, B**), which was highly suspected to be peripheral lung cancer. The Chest CT reveals multiple nodules of varying sizes(varying size range, 0.2-1.0 cm) are present in the upper and lower lobes of both lungs. The number of these nodules is approximately over 30 (**Figures 1C–F**). The hilar and mediastinal lymph nodes were not enlarged (**Figure 1**). Laboratory examination as following: Whole blood leukocytes, 6.03x10^9^/L [neutrophils, 64.4% (normal range, 50-70%); lymphocytes, 28.2% (normal range, 20-50%); monocytes, 5% (normal range, 3-10%); eosinophils, 1.7% (normal range, 0.4 -8%); basophils, 0.7% (normal range, 0-1%)]; red blood cells, 3.78x10^12^/L (normal range, 4.3 to 5.8x10^12^/L); hemoglobin, 120 g/L (normal range, 115-150 g/L); platelets, 243x10^9^/L (normal range, 125-350x10^9^/L); blood glucose, 4.67 mmol/L (normal range, 3.9-6.1 mmol/L); total bilirubin, 10.31 µmol/L (normal range, 3.42-20.5 µmol/L); aspartate aminotransferase, 17.3 U/L (normal range, 0-40 U/L); alanine aminotransferase, 11.4 U/L(normal range, 0-50 U/L); lactate dehydrogenase, 102 IU/L (normal range, 100-240 IU/L); highly sensitive C reactive protein, 1.52 mg/L (normal range, 0-5 mg/L); and Erythrocyte Sedimentation Rate, 5 mm/h (normal range, 0-20 mm/h). A urinalysis and microscopic examination were normal. Tumour markers were as follows: Neuron specific enolase, 11.9 ng/mL (normal range, 0-16.3 ng/mL); carcinoembryonic antigen, 0.8 µg/L (normal range, 0-5 µg/L); and ferritin, 67.1 ng/mL (normal range, 30-400 ng/mL), all of which were at normal levels. Sputum Gram staining and bacterial culture showed no microorganisms. Acid fast staining and sputum culture showed no acid-fast bacteria. Bronchofiberscopy showed no lesions in the trachea and bronchus. Bacterial, cytological, and pathological examinations from the bronchoscope showed negative results.

**Figure 2:**
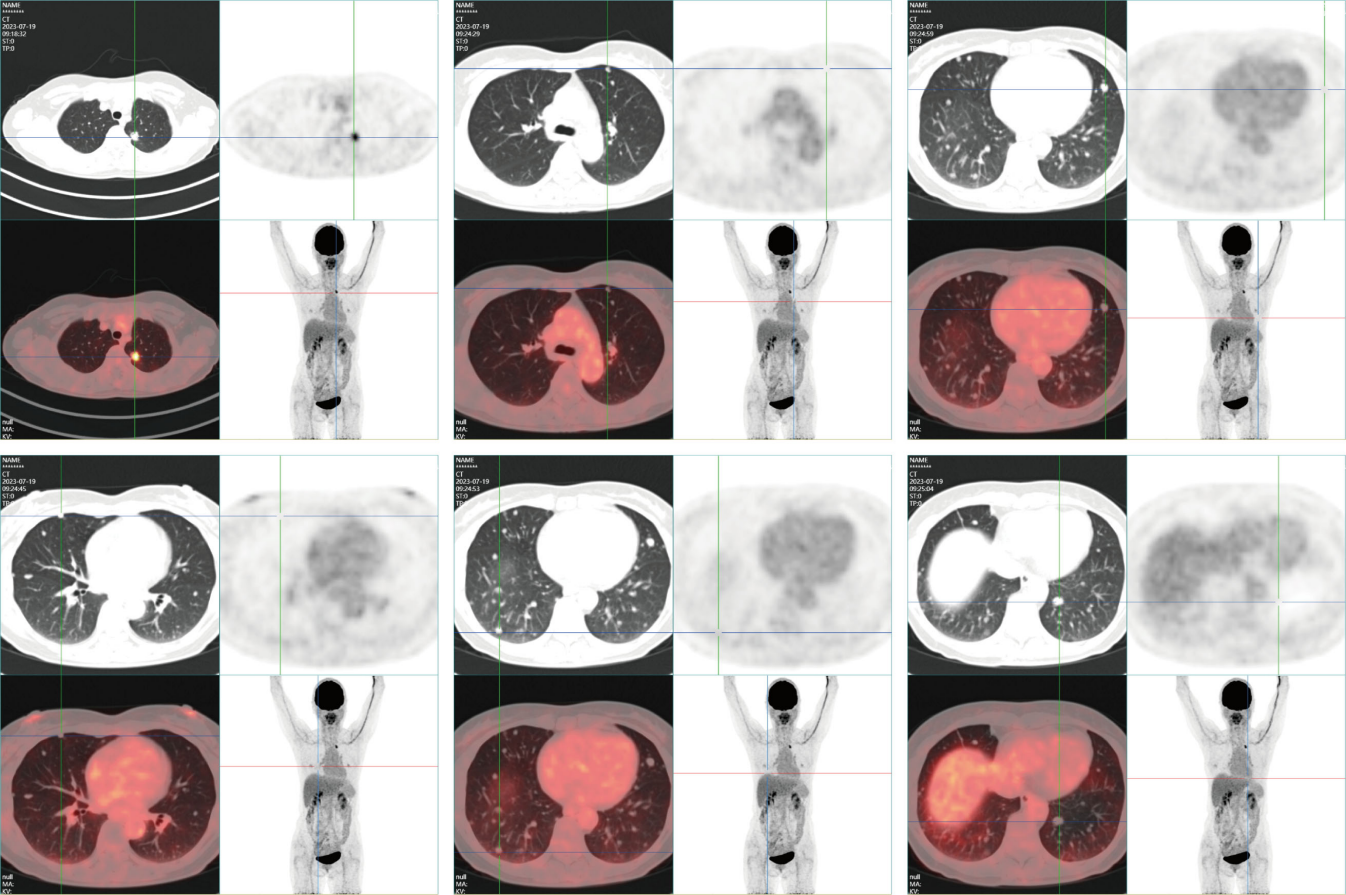
PET-CT reveals an irregular nodule subpleurally in the apicoposterior segment of the left upper lobe, measuring approximately 1.4x1.3 cm. The nodule, adorned with minute spiculations, exerts traction on the adjacent pleura and exhibits abnormal radiotracer accumulation, with an SUVmax of 7.1. Multiple variably sized, quasicircular nodules are identified in both lungs, with diameters ranging from approximately 0.2-1.0 cm; these do not exhibit abnormal radiotracer concentrations. No additional abnormal solid changes or radiotracer accumulations are observed. No evident enlargement or abnormal radiotracer uptake was identified in the mediastinal lymph nodes.

A Whole-body ^18^F-FDG PET-CT on July 19, 2023 revealed a density anomaly in the nodule in the apicoposterior segment of the upper left lobe complicated by elevated glucose metabolism with a maximum standardised uptake value (SUVmax) of 7.1 and no significant elevated glucose metabolism of the other multiple nodules in either lung with an SUVmax of 0.3-0.5 (**Figure 2**), including those nodules that were larger than 8 mm.

**Figure 3:**
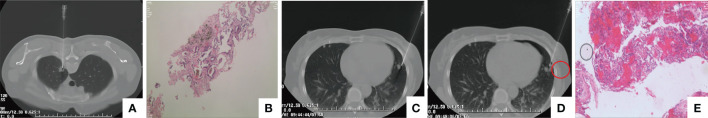
CT-guided percutaneous needle biopsy. **(A)** Percutaneous biopsy of the apicoposterior segment nodule in the left upper lobe. **(B)** Biopsy of the upper left lobe confirming adenocarcinoma of pulmonary origin (HE, ×100, scan view). **(C)** The first needle biopsy of a nodule in the lingular segment of the left upper lobe. **(D)** The second needle biopsy of the same nodule, with a visible haemorrhagic focus at the biopsy site (within the red circle). **(E)** The biopsied tissue from the lingular segment of the upper left lobe, revealing haemorrhage and minor fibrous exudation within the alveolar cavity under the microscope. Focal widening of the alveolar septa and reactive hyperplasia of alveolar epithelial cells were noted, with no evident cellular atypia (HE, ×100; scan view).

**Figure 4:**
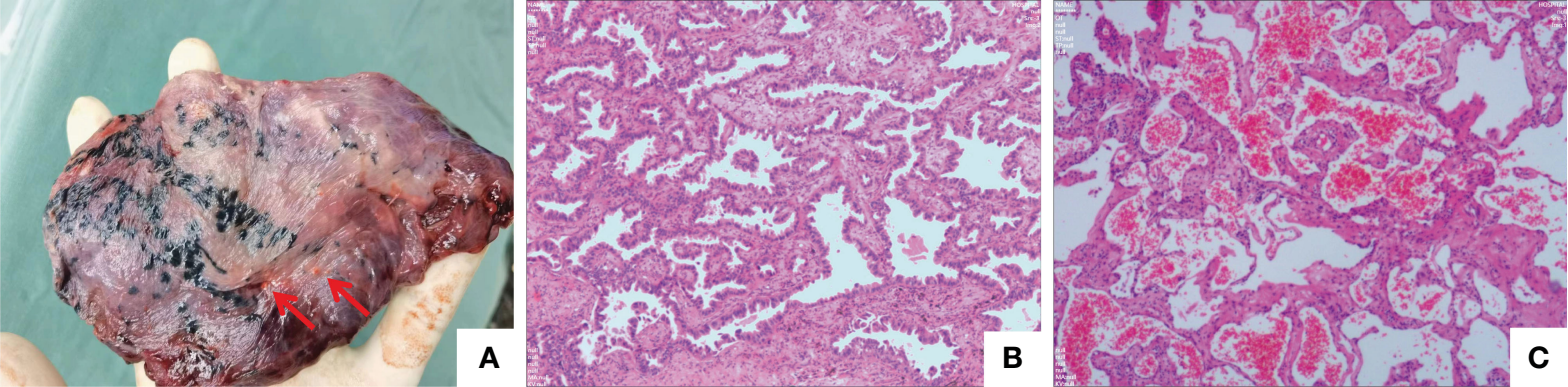
Surgical pathology. **(A)** The resected left upper lobe. The nodules identified by the two red arrows are atrophic cavernous haemangiomas. **(B)** This lesions was an invasive adenocarcinoma, predominantly characterised by an acinar growth pattern. The tumour dimensions were 1.8 cm x 1 cm x 0.8 cm, and there was no involvement of the pleura or bronchi or evidence of vascular invasion or perineural invasion. Tumour spread through air space (STAS) is observed. **(C)** Another examined nodule in the upper left lobe, pathologically diagnosed as a cavernous haemangioma with a diameter of 0.8 cm (HE, ×200; scan view). Immunohistochemical staining yielded CD31(+), CD34(+), CK7(-), NapsinA(-), TTF-1(SPT24)(-), D2-40(-), and SMA(+), indicating a vascular origin.

**Figure 5:**
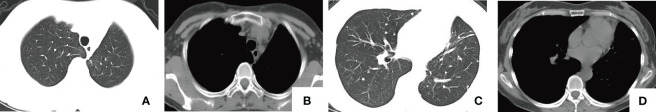
Four-months postoperative chest CT. **(A, B)** The postoperative state following the postoperative state following the lobectomy of the left upper lobe. **(C, D)** Multiple nodules of varying sizes in both lungs, with the larger ones measuring approximately 1.3 cm in diameter, consistent with previous findings.

To obtain a definitive diagnosis, a CT-guided percutaneous needle biopsy was performed on nodule in the apicoposterior segment of the upper left lobe (**Figure 3A**). Rapid On-Site Evaluation (ROSE) indicated adenocarcinoma. Subsequently, hematoxylin-eosin (HE) staining of biopsy specimens confirmed to be primary lung adenocarcinoma (**Figure 3B**), the immunostaining revealed positive results for TTF-1 (+), NapsinA (+), CK7(+). However, to determine whether the nature of other nodules is intrapulmonary metastasis, considering that the nodule in left lingual lobe is close to the adenocarcinoma nodule and the nodule size is relatively large, a subsequent percutaneous lung biopsy was performed on the nodule in left lingual lobe (**Figures 3C, D**) on July 28, 2023. Postoperative pathology showed haemorrhage and a small amount of fibrous exudation in the alveolar cavity, focal widening of the alveolar septum, and reactive hyperplasia of alveolar epithelial cells with no obvious atypia (**Figure 3E**). The Multiple Disciplinary Team (MDT) concluded that a definitive diagnosis of malignant tumour in the apicoposterior segment of the left upper lobe was obtained, meanwhile, PET-CT suggested that other nodules were benign and the biopsied nodule in left lingual lobe was benign also, therefore, insufficient evidence of metastatic tumours was found for the other multiple nodules in both lungs, and resection of some nodules followed by pathological examination was recommended. On August 2, 2023, the patient underwent video-assisted thoracoscopic surgery (VATS) for the left upper lobe. Intraoperative exploration revealed no pleural effusion or adhesions, poorly developed interlobar fissures, and multiple small nodules in the upper and lower lobes of the left lung. A wedge resection of some nodules in the left upper lobe was performed, and intraoperative frozen section indicated benign tumours. Macroscopically, the wedge-resected lung tissue was 3.3x2.5x1-cm in size, and a nodule with a diameter of 0.8x0.6x0.8-cm was seen in its section. The section was gray-white, gray-black, solid, medium in texture, with unclear boundary with the surrounding area and adjacent to the pleura. Other lung tissue sections are gray-red and soft. Histopathologically, cavernous haemangioma was considered, and immunostaining suggested CD31(+), CD34(+), CK7 (-), NapsinA(-), TTF-1(SPT24)(-), CD34(+), CK7 (-). D2-40(-), SMA(+)(**Figure 4C**). Lobectomy for the lung cancer in the left upper lobe was performed, including a dissection of the lymph nodes in groups 5 (2 nodes), 6(1 node), 11(3 nodes), and 12 (4 nodes), as well as the hilar (1 node), interlobar (1 node), and subcarinal lymph nodes (2 nodes). Macroscopic examination revealed that the excised 15x9x21−cm upper left lobe included a 1.8x1x0.8−cm mass with gray and grayish black sections, solid, medium in texture. Most of the mass was separated from the surrounding lung and partially attached to the pleura, which was not well-defined from the surrounding boundary. The mass was adjacent to the pleura and did not involve the bronchus (**Figure 4A**). Histologically, the tumour cells were moderately differentiated invasive adenocarcinoma of the acinar and micropapillary type, tumour spread through air space (STAS) was observed in the surrounding alveolar space (**Figure 4B**), no metastatic cancer was found in the lymph nodes (0/17), the pathological TNM stage of the lung cancer was T1_b_N_0_M_0_. The final diagnosis was pulmonary adenocarcinoma coexisting with diffuse pulmonary cavernous haemangiomas. The patient was discharged on the fourth postoperative day without complications. Genetic molecular testing for EGFR or KIT mutations using PCR-Sanger sequencing was performed by the Clinical Molecular Diagnostic Center of Taihe Hospital, Hubei University of Medicine, and an *EGFR* (T790M/L858R) mutation was reported. Osimertinib (80 mg orally once daily) was subsequently administered, and following 4 months of therapy, a chest contrast−enhanced CT revealed multiple nodules in both lungs, which were almost unchanged compared with before (**Figure 5**). The patient was followed up, 4 months to date, and remained in good health with no evidence of recurrence. Meanwhile, regular follow−up of the patient is ongoing.

## Discussion

PCHs are benign tumours caused by vascular malformations in the lungs. Cavernous haemangiomas usually occur in the skin, subcutaneous tissue, and muscles, but can also appear in the liver, kidneys, bones, and bladder; However, cavernous haemangiomas in the lungs are rare (3). Radiologically, lesions of PCHs can be isolated or multiple; diffuse pulmonary cavernous haemangiomas may be associated with hereditary haemorrhagic telangiectasia (HHT), which is a rare autosomal dominant genetic disorder characterised by recurrent epistaxis, cutaneous and mucosal telangiectasias, and arteriovenous malformations in visceral organs, including the liver, lungs, brain, and gastrointestinal tract (4). The pathogenesis is mainly associated with mutations in the ENG, ACVRL1 (also known as ALK1), and Smad4 genes (5). In this case, the patient was a rare bilateral diffuse cavernous haemangioma, and the possibility of HTT should be considered. However, the patient had no related symptoms or family history. PCHs can occur at any age but are most prevalent among individuals aged 40 to 60, with a male-to-female incidence ratio of approximately 1:3, and they are more commonly located in the right lung. The clinical manifestations of PCHs are often nonspecific. Patients might be asymptomatic, and pulmonary lesions are usually found incidentally during a routine physical examination, or patients present with symptoms such as cough, expectoration, hemoptysis, chest discomfort, chest pain, sudden respiratory distress, and intrathoracic bleeding. The clinical manifestations are correlated with the location, size, number, and whether vascular degeneration has occurred within the cavernous haemangiomas (6).

Due to the rarity of PCHs and the lack of specificity in most auxiliary examinations, misdiagnoses are common, often confusing PCHs with other lung diseases, such as lung cancer, tuberculosis, inflammatory pseudotumours, lung cysts, pulmonary sequestration, and hamartoma (7). Chest X-rays typically show round or oval masses with smooth, well-defined edges and uniform density. Radiologically, the density of the lesion was similar to that of the adjacent soft tissue, with phlebolith formation indicating a characteristic presentation of the disease, possibly due to intravenous thrombosis accompanied by calcium salt deposition. Contrast-enhanced CT reveals rapid lesion enhancement, with CT values often usually between 30-60 HU, maintaining isodensity or slightly hyperdensity during delayed scanning. However, some lesions may not be well enhanced due to the absence of large supplying arteries or the presence of significant arteriovenous shunt (8). In this case, PET/CT might offer significant assistance in diagnosing PCHs, in general, which do not show abnormally high uptake of ^18^F-FDG and 11C-choline (two common oncologic radiotracers), enabling distinction from malignant tumours and certain pulmonary inflammatory lesions that exhibiting increased uptake (9). Bronchoscopy may identify haemangiomas that project into the bronchial lumen. Caution should be exercised when considering a biopsy of a suspected haemangioma as it has extensive connections to adjacent blood vessels and can lead to uncontrolled bleeding which, if not treated immediately, can lead to potential suffocation and death. Percutaneous needle biopsy (PCNB) should also be performed with caution, as puncture haemangiomas can lead to severe hemopneumothorax, endangering the patient’s life (10). In this case, percutaneous needle biopsy procedures were conducted for PCHs lesion located in the lingular segment of the left upper lobe. During biopsy procedure, mild bleeding was observed at the puncture site (**Figure 3D**), and the patient also experienced mild haemoptysis after surgery. Fortunately, severe haemorrhage was not observed, which was probably due to the biopsy site located in the peripheral lung, where the vascular density is low, and with small blood vessels, in addition, the patient’s coagulation function was normal. Overall, the biopsy of the PCHs lesion in the left lingual lobe in this case was ultimately due to its rarity. If potential diagnosis of PCHs has been considered preoperatively, biopsy might have been avoided. This case provides some references for future clinical practice. In addition, the histopathology of a percutaneous lung biopsy showed only intrapulmonary bleeding without a definitive diagnosis. This suggests that there is limitation for percutaneous lung biopsy in the diagnosis of PCHs, even if enough tissue specimens are obtained, the specimens are often inadequate to meet the diagnostic requirements, which suggesting that PCNB should not be the primary diagnostic approach for PCHs.

The definitive diagnosis of PCHs depends on postoperative histopathological findings. Grossly, they have no capsule, with a reddish-brown surface, and exhibit a honeycomb appearance on the section. Microscopically, the tumour consists of dilated vascular sinuses or intercommunicating capillaries lined by endothelial cells, accompanied by minimal fibrous tissue septa and lymphocytes (11). Since PCHs is a benign tumour originating from the vascular endothelium, immunohistochemical staining of endothelial markers such as CD31 and CD34 usually shows positive results. Staining for smooth muscle actin (SMA) may also be positive, whereas expression of TTF-1, an indicator of pulmonary malignancy is negative. The current treatment for PCHs based on the stage, size of the lesion, and the patient’s symptoms. For early-stage, small, asymptomatic, and definitively diagnosed PCHs, close follow-up can be performed. However, surgical intervention should be considered in cases where symptoms are present or the diagnosis is ambiguous and malignancy cannot be excluded (as in this case) (12).Alternatively, surgical treatment combined with surgical biopsy of lesions of unknown nature may contribute to establish the diagnosis. PCHs with large lesions are at a risk of spontaneous rupture, resulting in severe bleeding or hemoptysis. Therefore, once diagnosed, timely surgical treatment is crucial (13). Given that cavernous haemangiomas have extensive connections with the surrounding vasculature, which may cause uncontrollable bleeding. In view of this, resection of the lesion alone is not advisable, and lobectomy or segmental resection with extended resection of the lesion should be considered to avoid severe bleeding (14). Of course, for small haemangiomas located in the pleura or peripheral lung, wedge resection may be considered. Intraoperative frozen section can help to avoid unnecessary traumatic lymph node dissection. Additionally, international literature has documented alternative methods for treating PCHs, including interventional vascular embolisation, radiation therapy, alpha-interferon, and antiangiogenic drugs (15, 16).

In summary, PCHs are rare benign tumours in the lungs, while lung cancer coexisting with diffuse pulmonary cavernous haemangioma is more uncommon. Radiological findings are usually multiple round or oval nodules in the lung. There are often difficulties in using conventional and contrast-enhanced CT to distinguish these from metastatic lung cancer, leading to misdiagnosis and delayed surgical treatment. PET-CT can be a valuable tool in centres with available resources. The normal metabolism of diffuse cavernous haemangiomas contrasts with the elevated glucose metabolism of lung cancer metastases, which may contribute to the diagnosis. However, the confirmed diagnosis still relies on pathological diagnosis. For the cases of early lung cancer complicated with PCHs, lung cancer can be surgically resected, and whether PCHs should be removed or not should be determined according to the size and distribution of the lesions, as well as relevant clinical symptoms of the patient.

## Data availability statement

The raw data supporting the conclusions of this article will be made available by the authors without undue reservation.

## Ethics statement

This study has been approved by Taihe Hospital ethics committee, and performed in accordance with the principles of Good Clinical Practice following the Tri-Council guidelines. Written informed consent was obtained from the patient for anonymized information to be published in this article.

## Author contributions

XW: Writing – original draft, Writing – review & editing, Conceptualization. TR: Data curation, Methodology, Writing – review & editing. HY: Formal analysis, Project administration, Supervision, Writing – review & editing. WH: Software, Validation, Writing – review & editing. JG: Conceptualization, Investigation, Writing – review & editing. MW: Conceptualization, Funding acquisition, Resources, Visualization, Writing – original draft.
